# Response to: 500 mg as bolus followed by an extended infusion of 1500 mg of meropenem every 8 h failed to achieve in one-third of the patients an optimal PK/PD against nonresistant strains of these organisms: is CRRT responsible for this situation?

**DOI:** 10.1186/s13613-020-00781-6

**Published:** 2020-12-04

**Authors:** Amol Kothekar, Jigeeshu Vasishtha Divatia, Sheila Nainan Myatra, Vikram Gota

**Affiliations:** 1 Department of Anesthesiology, Critical Care and Pain, Tata Memorial Centre, Homi Bhabha National Institute, Mumbai, India; 2 Department of Clinical Pharmacology, ACTREC, Tata Memorial Centre, Homi Bhabha National Institute, Kharghar, Navi Mumbai, India

We thank Prof. Honore and colleagues for their interest in our article [1] and their thought-provoking comments [2].

They are indeed correct in observing that in our study pertaining to the patients with severe sepsis or septic shock, 3 h extended infusions (EI) of 1000 mg of meropenem, administered every 8 h failed to achieve a fraction of time (fT) > 4 μg/mL > 40 in more than one-third of patients. We were, therefore, intrigued by their title “500 mg as bolus followed by an extended infusion of 1500 mg of meropenem every 8 h failed to achieve in one-third of the patients an optimal PK/PD against nonresistant strains of these organisms”. We believe that their title needs to be corrected as the results of our modelling clearly showed that 500 mg as bolus followed by an extended infusion of 1500 mg of meropenem every 8 h would achieve fT > 4 μg/mL > 40 effective against non-resistant strains of these organisms in all patients.

They also raise an important question regarding the proportion of patients in the study who received continuous renal replacement therapies (CRRT), as meropenem is known to have significant elimination by CRRT [3].

None of our patients during the study period of 72 h required or were subjected to any form of renal replacement therapy including CRRT. Therefore, it follows that the lower exposure observed in eight out of 24 (33%) patients in our study was due to an inherent alteration in the disposition of meropenem in our cohort of patients and not due to extraneous factors such as CRRT.

We would like to note that the observations made by Isla et al. may not apply in our case due to inherent difference in study population [1, 3]. Patients in the study by Isla et al. had higher APACHE II scores (19.4 ± 6.8 vs 15.4 ± 8.09) and SOFA scores (13.1 ± 4.0 vs 7.35 ± 3.62) compared to our study population. Moreover, their patients had lower calculated creatinine clearance (CL_CR_) (37.4 ± 42.3 mL/min vs 73.8 ± 26.6 mL/min) and frequent need of CRRT (50% of patients). We excluded patients with baseline calculated creatinine clearance (CL_CR_) < 50 mL/min and those not expected to survive for 72 h.

To conclude, lower exposure of conventional 3 h of extended infusions of meropenem in adult patients with severe sepsis and septic shock in our study indicates altered natural disposition of meropenem rather than elimination of meropenem by CRRT.

## Data Availability

The datasets used and/or analyzed during the current study are available from the corresponding author on reasonable request.
